# Role of Granulosa Cells in the Aging Ovarian Landscape: A Focus on Mitochondrial and Metabolic Function

**DOI:** 10.3389/fphys.2021.800739

**Published:** 2022-01-27

**Authors:** Hannah C. Alberico, Dori C. Woods

**Affiliations:** Department of Biology, Northeastern University, Boston, MA, United States

**Keywords:** granulosa cells, ovarian aging, mitochondria, reproduction, oocytes

## Abstract

Mitochondria are at the intersection of aging and fertility, with research efforts centered largely on the role that these specialized organelles play in the relatively rapid decline in oocyte quality that occurs as females approach reproductive senescence. In addition to various roles in oocyte maturation, fertilization, and embryogenesis, mitochondria are critical to granulosa cell function. Herein, we provide a review of the literature pertaining to the role of mitochondria in granulosa cell function, with emphasis on how mitochondrial aging in granulosa cells may impact reproduction in female mammals.

## Introduction

Mitochondria are bacterial in origin with an ancestral genome descended from *Alphaproteobacteria*, which incorporated into the cytoplasm following endosymbiosis over 1.45 billion years ago (Gray, 2012). Subsequently, the internalized bacterial genome evolved into the mitochondrial genome, and the ubiquitous organelle is now a critical participant in eukaryotic cellular function. Mitochondria harbor their own genetic material (mtDNA), distinct from the nuclear genome (nDNA), which in mammals is nearly exclusively inherited through the maternal germline, with rare instances of heteroplasmy as a result of paternal inheritance reported (Mitchell, 1961; Huttemann et al., 2008; Perry et al., 2011; Luo et al., 2018; Annis et al., 2019). The circular genome of the mitochondrion is small—in humans only approximately 16 kb—and encodes for 37 genes, including 13 proteins associated with the subunits of the electron transport chain (ETC), as well as 2 ribosomal RNAs (rRNAs) and 22 transfer RNAs (tRNAs). The remaining gene products associated with mitochondria, including those used for ETC function, are nuclear encoded. Thus, 99% of the mitochondrial proteome emanates from the nucleus, highlighting the degree in which mitochondrial: nuclear communication through antegrade and retrograde signaling work in concert. Notably, while mitochondria are well characterized for their role in cellular bioenergetics, these organelles are highly specialized based on tissue- and cell-type, and perform additional functions based on specific cellular demands. These include critical roles in apoptosis, thermogenesis, heme biosynthesis, detoxification, calcium signaling and ion flux, and steroidogenesis, among others (Baughman et al., 2011; Friedman and Nunnari, 2014; Woods, 2017).

**FIGURE 1:**
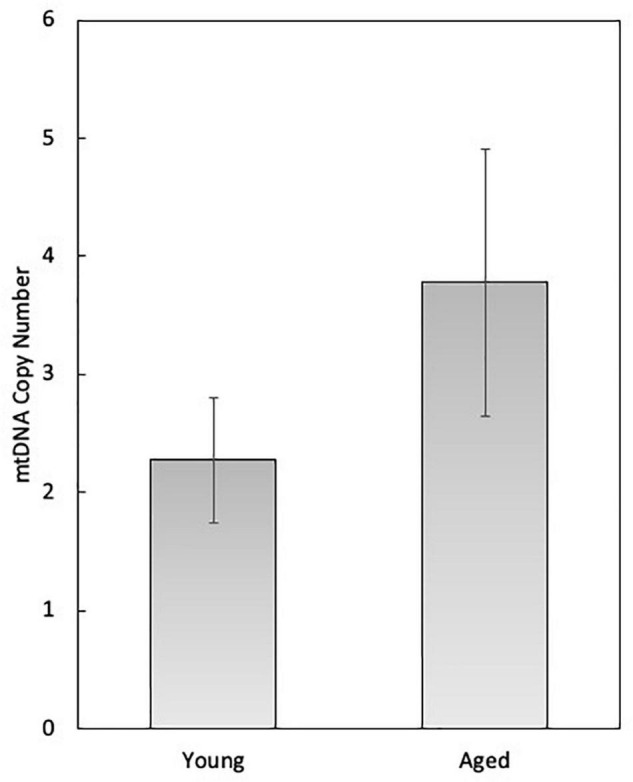
Single-molecule PCR (smPCR) analysis of oocyte mitochondria collected from 6-week-old (young) and 12-month-old (aged) C57BL/6 mice for the determination of mtDNA copy number on a per-organelle basis. The average mtDNA copy number of the young mouse oocyte mitochondria was 2.27 (SEM = 0.53; *n* = 22), and of aged mice was 3.27 (SEM = 1.13, *n* = 27).

In female mammals, mitochondrial function has been studied in detail in the granulosa and theca cells of the developing follicle, as well as in the oocyte and developing embryo. During follicle development, mitochondria within the oocyte undergo substantial numerical expansion through mitochondrial biogenesis, while mechanisms for mitochondrial autophagy (e.g., mitophagy) are latent until the 4–8 cell stage of embryogenesis (Tsukamoto et al., 2008; Boudoures et al., 2017). At the time of ovulation, mitochondrial biogenesis ceases and does not resume until the blastocyst stage, around the time of implantation. Thus, during each cell division mitochondrial content and mtDNA copy number are reduced on a per-cell basis, and must maintain a critical threshold number (e.g., 40,000–50,000 copies of mtDNA) for successful development to the blastocyst and subsequent implantation (Wai et al., 2010). It has been estimated in oocytes that on a per-mitochondrion basis each organelle harbors 1–2 copies of mtDNA (Piko and Matsumoto, 1976), although this has yet to be empirically determined. Though slightly higher, our analysis of single oocyte mitochondria collected from both young (6-weeks old) and aged (12-months old) female mice by single-molecule PCR (smPCR) is in accordance with this estimation (2.27 ± 0.53 in young samples, 3.27 ± 1.13 in aged samples; N.S) (Figure 1). However, we have observed that smPCR of oocyte mtDNA produces negative results more frequently than mitochondria of other cell types (data not shown). Since we do not know the biological significance of this (if any), mitochondrial samples that were determined to have zero mtDNA copies in this experiment have been excluded from analysis, potentially leading to artificial elevation of the values reported in Figure 1.

Importantly, the endocrine function of the ovary is dependent upon the ovarian follicle, which synchronizes with the hypothalamic-pituitary-gonadal (HPG) axis (reviewed in Truman et al., 2017). Localized primarily within the ovarian cortex, ovarian follicles are considered the functional units of the ovary and are comprised of an oocyte surrounded by granulosa cells or, at the resting primordial phase, pre-granulosa cells, and enclosed within an extracellular matrix (ECM)-rich basement membrane. Research in rodent models has demonstrated that the pre-granulosa cells enclosed within primordial follicles are largely non-proliferative, although mitotically active pre-granulosa cells can be observed within primordial follicles of the medulla (Hirshfield and DeSanti, 1995). As follicles mature, granulosa cells transition from a squamous single layer to a cuboidal and multilaminar proliferative phenotype, and a theca layer is recruited. Together, the somatic cells of the follicle respond to circulating gonadotropins (i.e., FSHR and LHR) to generate the sex steroids (i.e., estrogens, progestins, and androgens), which is a mitochondrial-dependent process. At ovulation, the granulosa cells immediately surrounding the oocyte, termed cumulus granulosa cells, remain with the ovulated egg, while the mural granulosa cells along with the theca cells are retained in the ovary and functionally differentiate into the corpus luteum (Smith et al., 1994). Thus, a growing follicle consists of four cell types—an oocyte, mural granulosa cells, cumulus granulosa cells, and the cells comprising the theca interna and theca externa. Although both the theca and granulosa cells participate in endocrine function, granulosa cells specifically work in concert with the oocyte through bi-directional communication working *via* paracrine factors as well as gap junction signaling, which ultimately results in the growth and maturation of fertilization-competent eggs (Eppig et al., 2002; Gittens et al., 2005; Kidder and Vanderhyden, 2010; Wigglesworth et al., 2013).

Coincident with advancing maternal age, the number of follicles within the ovary wanes, and concurrently the quantity and quality of fertilization-competent eggs declines. The decline in oocyte quality is, at least in part, attributed to impaired mitochondrial function (reviewed in Woods et al., 2018). While many endpoints have been used to demarcate egg quality in the context of maternal aging, mitochondrial derangement and abnormal chromosomal segregation are repeatedly and persistently cited as instigators of developmental incompetence and embryonic failure (Henderson and Edwards, 1968; Hook, 1981; Hassold and Chiu, 1985; Battaglia et al., 1996; Tarin et al., 2001; Eichenlaub-Ritter et al., 2004; Hunt and Hassold, 2008; Pan et al., 2008; Selesniemi et al., 2011; Faraci et al., 2018; Woods et al., 2018). Highlighting the impact of mitochondrial function on the reproductive potential of oocytes with age, interventions aimed at modulating pathways associated with mitochondrial homeostasis result in several benefits, including improved spindle assembly, chromosomal segregation, and fertility outcomes (Tarin et al., 2002; Bentov et al., 2011; Selesniemi et al., 2011; Woods and Tilly, 2015; Liu et al., 2018; Bertoldo et al., 2020).

In addition to the well-characterized role of mitochondrial dysfunction with age on oocytes, the mitochondria of granulosa cells also reflect age-associated abnormalities. Complications in mitochondrial processes and function include a decrease in mitochondrial DNA (mtDNA) copy number, modifications in ultrastructure, increase in the frequency of mtDNA deletions and mutations, altered mitochondrial membrane potential (Δψ_m_) and metabolic function, and a reduced capacity for steroid hormone biosynthesis, among others (Tatone et al., 2011; Liu et al., 2017; MacDonald et al., 2017; Sreerangaraja Urs et al., 2020). Accordingly, it has been postulated that granulosa cell mitochondria undergo changes with age that can impart negatively upon oocyte quality and function. Similarly, it has been proposed that granulosa cell metabolites and quantifiable mitochondrial features can serve as molecular biomarkers for oocyte competence (Tatone et al., 2011; Boucret et al., 2015; Ogino et al., 2016; Desquiret-Dumas et al., 2017; Liu et al., 2017; MacDonald et al., 2017; Sreerangaraja Urs et al., 2020). Herein, we review the role of mitochondria in granulosa cells, with a focus on how mitochondrial function and dynamics within granulosa, cumulus, and luteal cells is altered with advancing maternal age.

## Mitochondria as Effectors of Granulosa Cell Function

Of the six classes of steroid hormones, three are synthesized in the ovary (Miller, 2013). In the two-cell model for steroidogenesis progestins and androgens are synthesized in the theca cells, and further converted to estrogens in the adjacent granulosa cells, with granulosa cells also producing progestins (Jamnongjit and Hammes, 2006). The process of steroidogenesis is dependent upon steroidogenic cell-specific mitochondria which harbor specialized enzymes required for steroid hormone biosynthesis. The role of granulosa and theca cells, as well as mitochondria, in steroid hormone production has been well studied and summarized elsewhere (Jamnongjit and Hammes, 2006; Miller, 2013; Yazawa et al., 2019). Specifically, in granulosa cells, mitochondria are critical for two processes integral to steroid hormone production; cholesterol import into mitochondria, and the enzymatic conversion of cholesterol into steroid hormones. The transfer of cholesterol from the cytoplasm into mitochondria is initialized following gonadotropin stimulation, and is facilitated by Steroid Acute Regulatory Protein (StAR), which interacts with a protein assembly including the 18 KDa translocator protein (TSPO) and the voltage-dependent anion channel (VDAC-1) on the outer mitochondrial membrane (OMM) to direct cholesterol to the inner mitochondrial membrane (IMM), where it is converted to pregnenolone and then progesterone, carried out by the mitochondrial enzymes CYP11a1 and 3-beta-hydroxysteroid dehydrogenase (HSD3β1), respectively (Kiriakidou et al., 1996; Miller, 2013). Not surprisingly, alteration of StAR and HSD3β1 protein levels have been linked to infertility (Feuerstein et al., 2007; Hamel et al., 2008; Wathlet et al., 2012; Sreerangaraja Urs et al., 2020). Furthermore, it is the transport of cholesterol into the IMM that is considered the rate-limiting step in steroidogenesis (Stocco, 2001), with functional mitochondria maintaining Δψ_m_ critical to this process (Artemenko et al., 2001; Allen et al., 2006; Miller, 2013, 2017; Sreerangaraja Urs et al., 2020).

Intact Δψ_m_ has been demonstrated to be requisite for steroidogenesis in Leydig cells (Allen et al., 2006), and been further associated with steroidogenic capacity of granulosa cells in bovine models (Ostuni et al., 2018). The pivotal role of mitochondria in modulation of steroidogenesis in granulosa cells has also been linked to fertility outcomes in women. In a recent study evaluating whether mitochondrial function is correlated with IVF outcomes, mitochondrial properties were evaluated in patient cohorts [e.g., endometriosis, ovarian endometrioma, endometriosis without ovarian endometrioma, and polycystic ovary syndrome (PCOS)], as compared to those undergoing IVF for male-factor infertility (Sreerangaraja Urs et al., 2020). Using flow cytometry for the quantitative evaluation of markers for mitochondrial function and quality in cumulus cells, including Δψ_m_ and mitochondrial mass, the authors determined that mitochondrial dysfunction is associated with a decrease in estradiol (E2), and is further linked to a global decline in fertility, including oocyte maturation and fertilization rates. Specifically, increased Δψ_m_ in cumulus cells was positively correlated with E2 content, whereas reduced mitochondrial mass was associated with infertility. These findings are consistent with previous work demonstrating marked reductions in Δψ_m_ in granulosa cells from women with PCOS, and, to a lesser extent, endometriosis (Karuputhula et al., 2013).

The reduction of steroid hormone biosynthesis with age is largely attributed to a decline in the number of ovarian follicles, and thereby a reduction in functional steroid-producing cells. It is no surprise then, that hallmarks associated with reproductive aging and senescence in women are those that are measurable based on a decline in granulosa cell activity, such as a decrease in circulating levels of anti-Müllerian hormone (AMH; produced by immature granulosa cells), reduced levels of estrogen (produced by mature granulosa cells), and elevated circulating levels of FSH (due to a lack of inhibin production by granulosa cells). These well-established clinical determinants of reproductive function have long-solidified the significant role of granulosa cells as indicators of the aging of the female reproductive tract. As a number of mitochondrial deficits are known to occur with age, it is possible that vast changes in mitochondrial properties may lead to age-associated granulosa cell dysfunction, including abnormal steroidogenesis. For example, in female patients undergoing IVF, Δψ_m_ is significantly reduced in patients ≥38 years of age (Liu et al., 2017), although an earlier study showed no difference with age (Muhammad et al., 2009). This discrepancy could potentially be attributed to the differences in methodology employed with the later study using a combination of quantitative endpoints, such as flow-cytometry and quantitative microscopy, and the earlier study relying on a qualitative assessment based on fluorescence imaging. Nonetheless, additional alterations at the level of the mitochondria discussed below may also impact the ability of aged granulosa cells to synthesize steroid hormones. Further work directly linking mitochondrial abnormalities specifically with altered steroidogenesis is needed.

## Bi-Directional Communication, Role for Gap Junctions in Follicular Metabolism

Follicle growth and development is dependent upon bi-directional communication between the oocyte and the surrounding granulosa cells, which occurs through paracrine and juxtracrine signaling, the latter of which is maintained *via* gap junctions (Eppig, 1979; Lopez-Schier and St Johnston, 2001; Gittens et al., 2005; Su et al., 2008; Saadeldin et al., 2015). Gap junctions are oligomeric structures comprised of connexins which enable direct intercellular communication. First described by Anderson and Albertini (1976), gap junctions between the granulosa cells and developing oocytes were visualized by lanthanum tracer and freeze-fracture electron microscopy (EM). Gap junction-mediated communication between adjacent granulosa cells is modulated by connexin-43 (Cx43) gap junctions, whereas gap junctions between the granulosa cells and the oocyte are homotypic for connexin-37 (Cx37), with Cx37 gap junctions creating a syncytial network between the two cell types (Kidder and Mhawi, 2002; Best et al., 2015). The oocyte has a low capacity for glucose metabolism, due in part to low phosphofructokinase activity (Cetica et al., 2002; Dumesic et al., 2015), while granulosa cells are largely glycolytic and supply pyruvate, along with amino acids and cholesterol, to the oocyte through Cx37 gap junctions (Anderson and Albertini, 1976; Gilula et al., 1978; Bornslaeger and Schultz, 1985). The mitochondria within the oocyte then convert the granulosa cell-derived pyruvate to acetyl CoA, which then enters the tricarboxylic acid (TCA) cycle and ETC to synthesize ATP (Sutton-McDowall et al., 2010). In a regulatory loop, the oocyte secretes paracrine factors, including Bone Morphogenetic Protein 15 (BMP15), Growth and Differentiation Factor 9 (GDF9), and Fibroblast Growth Factor 8 (FGF8), which coordinate to promote glycolysis in granulosa cells (Sugiura et al., 2007). In addition to resource sharing and maintenance of the oocyte–granulosa regulatory loop, diffusion of cGMP through Cx37 gap junctions of blocks the resumption of meiosis in immature follicles (Pincus and Enzmann, 1935; Downs and Eppig, 1987; Tornell et al., 1991; Carabatsos et al., 2000; Norris et al., 2009). Furthermore, it has recently been demonstrated using porcine cumulus-oocyte-complexes (COCs) that cumulus cells can utilize Cx37 gap junctions to deliver ATP to oocytes (Kansaku et al., 2017).

**FIGURE 2:**
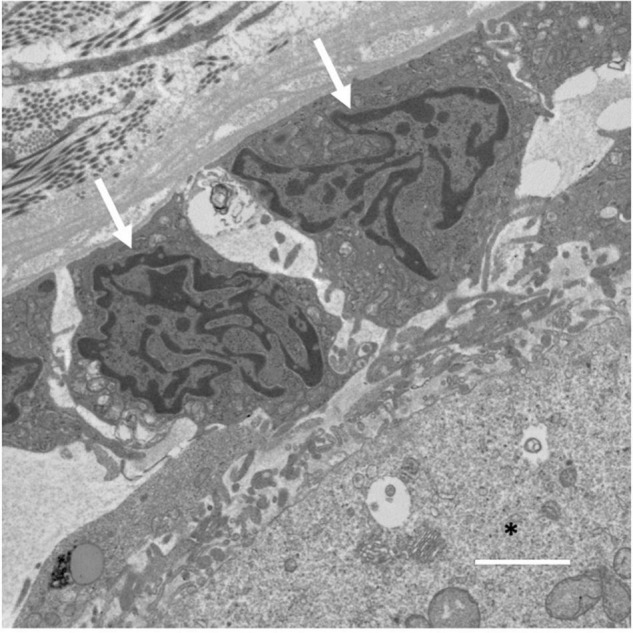
Electron micrograph of an ovarian follicle at the oocyte-granulosa cell interface, with the oocyte identified using a black asterisk, and two granulosa cells identified with white arrows. Scale bar represents 2 μm.

Gap junction communication between the oocyte and granulosa cells is made possible through transzonal projections (TZPs), which originate as filopodia from granulosa cells and process into the zona pellucida surrounding the oocyte (El-Hayek et al., 2018). An electron micrograph of the granulosa cell-oocyte interface where TZPs are found has been included for reference (Figure 2). TZPs are not restricted to the granulosa cells immediately surrounding the oocyte, as imaging demonstrates that granulosa cells in distal layers extend long, actin-rich filaments to the oocyte (El-Hayek et al., 2018). Strikingly, recent analysis of Cx43 and Cx37 gap junctions on high pressure-frozen ovarian tissue using three-dimensional EM and immunogold detection has revealed gap junction internalization *via* connexosomes (Norris, 2021; Norris and Terasaki, 2021). Moreover, within Cx43 connexosomes mature organelles, including mitochondria and endosomes are visibly observable, indicating cell-to-cell movement of organelles within the granulosa cell layer (Norris, 2021). Mitochondria can also be observed in deeply invaginated TZPs with Cx37-labeled gap junctions protruding into oocytes, implying the potential for mitochondrial transfer between granulosa cells and oocytes (Norris and Terasaki, 2021). Regulation of TZP formation by granulosa cells is facilitated by GDF9, one of the most well-characterized paracrine factors generated by oocytes. GDF9, signaling through the SMAD signaling pathway, upregulates the TZP-associated genes *fascin homolog 1* (*Fscn1*), an actin bundling protein and *myosin X* (*Myo10*), an actin-based motor protein, both known to induce formation of filopodia (Bohil et al., 2006; Hashimoto et al., 2011; El-Hayek et al., 2018; Baena and Terasaki, 2019). With age, oocyte GDF9 expression declines (Li et al., 2014; Park et al., 2020; Gong et al., 2021) along with the expression of *Fscn1* and *Myo10*, and the numbers of TZPs are reduced by approximately 40%, resulting in a marked decrease in gap junctional communication between the oocyte and the granulosa cells (El-Hayek et al., 2018).

Recent work demonstrates a role for the OMM GTPase, mitofusin1 (MFN1) in regulation of follicle development and maintenance of the follicle reserve, which occurs, at least in part, though maintaining Cx43 and Cx37 gap junctions (Zhang et al., 2019). In mice null for *Mfn* (*Mfn^–/–^*), mRNA expression of both *Cx37* and *Cx43* were significantly decreased in oocytes and granulosa cells. A combination of both fluorescence microscopy and EM revealed a significant reduction in gap junctions and transzonal projections (TZPs), which, along with a reduction in expression of E-cadherin and N-cadherin, significantly impaired communication between the granulosa cells and the oocyte, inhibiting growth past the primary stage of development. Notably, *Mfn^–/–^* mice showed an accumulation of ceramide, a membrane sphingolipid which accumulates on the OMM and is known to induce germ cell apoptosis, within oocytes (Perez et al., 2005). In aged mice, ceramide is trafficked through Cx37 gap junctions and plays a role in age-associated increased germ cell death through BCL2 Associated X, Apoptosis Regulator (Bax)-mediated apoptosis (Perez et al., 2005), a proapoptotic member of the B-cell lymphoma (Bcl-2) family of pro- and anti- apoptotic factors associated with mitochondrial apoptosis (Tilly et al., 1997). Importantly, treatment with myriocin to inhibit *de novo* ceramide synthesis in *Mfn^–/–^* mice improved the growth of secondary follicles and formation of antral follicles, leading to a partial rescue of the reproductive phenotype (Zhang et al., 2019), indicating that in addition to mitochondrial-specific effects on gap junction communication, metabolic by-products associated with altered mitochondrial function may be important targets to improve oocyte quality.

As the oocyte is considered the orchestrator of oocyte–granulosa cell communication, guiding the granulosa cells to produce factors to fulfill its needs throughout growth and folliculogenesis, it is perplexing why oocyte originating signals to sustain TZPs would decline. Perhaps this is to mitigate damage from the aging somatic environment, as aging granulosa cells may also have an impact on the decline in oocyte quality. Previous studies have collectively demonstrated that removal of the granulosa cell layer in aged female mice can have a positive impact on oocyte health and function. For example, Perez and Tilly demonstrated that removal of the granulosa/cumulus cell layer from eggs significantly reduced rates of apoptosis, essentially resulting in oocytes with a “youthful” phenotype (Perez and Tilly, 1997). How oocytes might be negatively influenced by the surrounding soma may involve circulating factors, the influence of an aging ovarian environment, as well as granulosa cells themselves.

## The Impact of Age on Granulosa Cell Mitochondria

**FIGURE 3:**
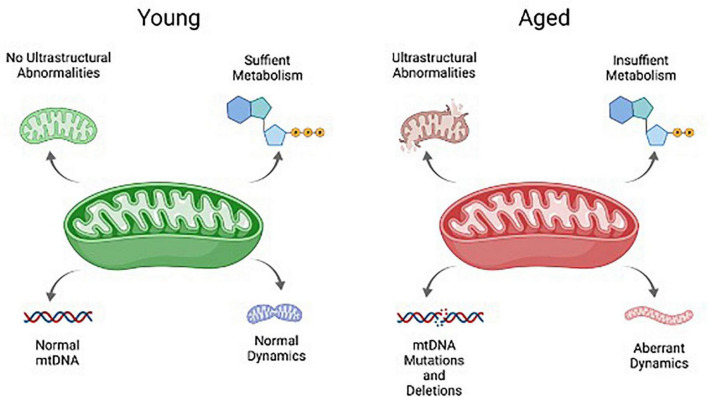
Schematic overview of key mitochondrial characteristics that are negatively impacted by aging. These include mtDNA, mitochondrial ultrastructure and integrity, metabolism, and mitochondrial dynamics.

Many of the hallmarks associated with aging mitochondria in the soma impact granulosa cells in women of advance maternal age or in animal models for ovarian aging. These include abnormalities in mitochondrial ultrastructure and integrity, metabolism, dynamics, and mtDNA mutations and deletions (Figure 3). Such features appear to be a function of age, and are not dependent upon follicle development, as abnormal mitochondrial ultrastructure can be found in even the resting follicles of aged women. An analysis of ovarian tissue obtained from women of advanced maternal age tellingly revealed a high frequency of ruptured mitochondrial membranes in the granulosa cells when compared to a younger cohort, indicative of increased mitochondrial destruction with age (de Bruin et al., 2004). As follicles grow and the granulosa cells expand, these abnormal mitochondrial features are exacerbated. An analysis of luteinizing granulosa cells obtained from follicular aspirates revealed destruction of mitochondrial membrane integrity, lack of cristae density, and vacuolization of the cristae and mitochondrial matrix with age (Tatone et al., 2006), while those obtained from a younger patient cohort had mitochondrial cristae with a tubular phenotype, which is notably associated with high steroidogenic activity (Rotmensch et al., 1986; Tatone et al., 2006).

The accrual of atypical mitochondrial ultrastructure in aged granulosa cells as they progress through follicle development may also be attributed to increasing reactive oxygen species (ROS), detectable in follicular fluid samples. With age, key genes associated with the neutralization of ROS, including *superoxide dismutase 1* (*SOD*1), *SOD2*, and *catalase*, are reduced, indicative of impaired ROS scavenging activity. This has important implications, as *SOD1* and *2* expression is elevated in response to oxidative stress and inflammation, both of which are associated with the ovulatory response (Espey, 1980), with anti-inflammatory medications reported to inhibit ovulation (Espey et al., 1982). Moreover, ovulation itself has been shown to be dependent upon LH-induced ROS production, with H_2_O_2_ capable of mimicking LH-induced cumulus expansion, and ablation of ROS blocking genes critical for ovulation (Shkolnik et al., 2011). It has been noted that the expression pattern of SOD1 is identical to that of HSD3β1 (Sugino, 2005), and that dehydrogenation of pregnenolone to form progesterone may be dependent upon SOD1 (Agrawal and Laloraya, 1977). In *Sod1*-deficient female mice, levels of progesterone fall far below those of WT mice, even following superovulation (Noda et al., 2012). Furthermore, superoxide significantly impairs Δψ_m_ in the absence of SOD1, resulting in severe mitochondrial damage (Aquilano et al., 2006), and is consistent with the reduced Δψ_m_ observed in women of advanced maternal age (Liu et al., 2017). Therefore, the declining levels of ROS scavengers with age may have profound impacts on granulosa cell function, from accelerated damage due to ROS to reduced steroidogenic capacity.

Additionally, the link between accumulating ROS and mtDNA damage, deletions, and mutations as they occur with age is well known (Loeb et al., 2005). It has been posited that mtDNA content of cumulus cells may be a non-invasive prognostic for embryo quality (Diez-Juan et al., 2015; Fragouli et al., 2015; Desquiret-Dumas et al., 2017), although this has been experimentally questioned (Victor et al., 2017). More recent evidence based on quantitative PCR to evaluate the ratio of mtDNA:nDNA indicates that mtDNA content of cumulus cells is negatively correlated with age, and further supports the finding that mtDNA content of cumulus cells may be considered as a biomarker for IVF outcomes (Yang et al., 2021). In addition to a decline in mtDNA copy number with age, the propensity for granulosa cells to acquire mutations and deletions with age has been examined. In a study evaluating the so called “common deletion” which is a 4,977 bp deletion of mitochondrial DNA (ΔmtDNA^4977^), with an early study concluded that the frequency of ΔmtDNA^4977^ in granulosa increases with age (Seifer et al., 2002). However, more recent work with a larger patient cohort suggests that age does not appear to be a factor in the frequency of this deletion in mural granulosa or cumulus cells (Au et al., 2005; Chan et al., 2006). The latter conclusion has been further supported, with recent evidence also indicating that while there does not appear to be a correlation between ΔmtDNA^4977^ and age, ΔmtDNA^4977^ is associated with granulosa cell apoptosis (Au et al., 2005). Advances in sequencing platforms, such as Long-molecule UMI-driven Consensus Sequencing (LUCS), will enable more detailed analysis of the mitochondrial mutations and damage that might occur with age (Annis et al., 2020).

## Conclusion

Granulosa cells are critical for ovarian function, including steroid hormone biosynthesis and as cooperative partners for oocyte growth and maturation. Therefore, it is not surprising that there has been an interest in evaluating the mitochondria of granulosa and cumulus cells as biomarkers for ovarian function, including oocyte and embryo quality. Intriguingly, recent evidence suggests cell-free mtDNA is released into the follicular fluid, presumably by granulosa or cumulus cells, and that this may potentially be used as a non-invasive biomarker for oocyte quality (Huo et al., 2020). Additional work on mitochondrial properties, dynamics, and function will likely reveal new and important details on the biological significance of these findings. Moreover, as technological advances in DNA sequencing and metabolomics continue to improve, further evaluation of how granulosa cells may act as effectors, positive and negative, of oocyte function can be addressed.

## Author Contributions

HA and DW wrote the manuscript. Both authors contributed to the article and approved the submitted version.

## Conflict of Interest

DW declares interest in intellectual property described in U.S. Patent 8,642,329, U.S. Patent 8,647,869, U.S. Patent 9,150,830, and U.S. Patent 10,525,086. The remaining author declares that the research was conducted in the absence of any commercial or financial relationships that could be construed as a potential conflict of interest.

## Publisher’s Note

All claims expressed in this article are solely those of the authors and do not necessarily represent those of their affiliated organizations, or those of the publisher, the editors and the reviewers. Any product that may be evaluated in this article, or claim that may be made by its manufacturer, is not guaranteed or endorsed by the publisher.
